# Nutrients in Energy and One-Carbon Metabolism: Learning from Metformin Users

**DOI:** 10.3390/nu9020121

**Published:** 2017-02-10

**Authors:** Fedra Luciano-Mateo, Anna Hernández-Aguilera, Noemi Cabre, Jordi Camps, Salvador Fernández-Arroyo, Jose Lopez-Miranda, Javier A. Menendez, Jorge Joven

**Affiliations:** 1 Unitat de Recerca Biomèdica, Hospital Universitari Sant Joan, Institut d’Investigació Sanitària Pere Virgili, Universitat Rovira i Virgili, 43201 Reus, Spain; fedra.luciano@gmail.com (F.L.-M.); anna.hernandeza@gmail.com (A.H.-A.); noemi.cabre@gmail.com (N.C.); jcamps@grupsagessa.com (J.C.); salvador.fernandez@iispv.cat (S.F.-A.); 2 Molecular Oncology Group, Girona Biomedical Research Institute (IDIBGI), 17190 Girona, Spain; 3 CIBER Fisiopatología Obesidad y Nutrición (CIBEROBN), Instituto de Salud Carlos III, 28029 Madrid, Spain; jlopezmir@uco.es; 4 ProCURE (Program against Cancer Therapeutic Resistance), Metabolism and Cancer Group, Catalan Institute of Oncology, 17190 Girona, Spain; 5 The Campus of International Excellence Southern Catalonia, 43003 Tarragona, Spain

**Keywords:** diabetes mellitus, energy intake, epigenetics, folic acid, food-drug interactions, food source, obesity, vitamins B

## Abstract

Metabolic vulnerability is associated with age-related diseases and concomitant co-morbidities, which include obesity, diabetes, atherosclerosis and cancer. Most of the health problems we face today come from excessive intake of nutrients and drugs mimicking dietary effects and dietary restriction are the most successful manipulations targeting age-related pathways. Phenotypic heterogeneity and individual response to metabolic stressors are closely related food intake. Understanding the complexity of the relationship between dietary provision and metabolic consequences in the long term might provide clinical strategies to improve healthspan. New aspects of metformin activity provide a link to many of the overlapping factors, especially the way in which organismal bioenergetics remodel one-carbon metabolism. Metformin not only inhibits mitochondrial complex 1, modulating the metabolic response to nutrient intake, but also alters one-carbon metabolic pathways. Here, we discuss findings on the mechanism(s) of action of metformin with the potential for therapeutic interpretations.

## 1. Introduction

**Figure 1:**
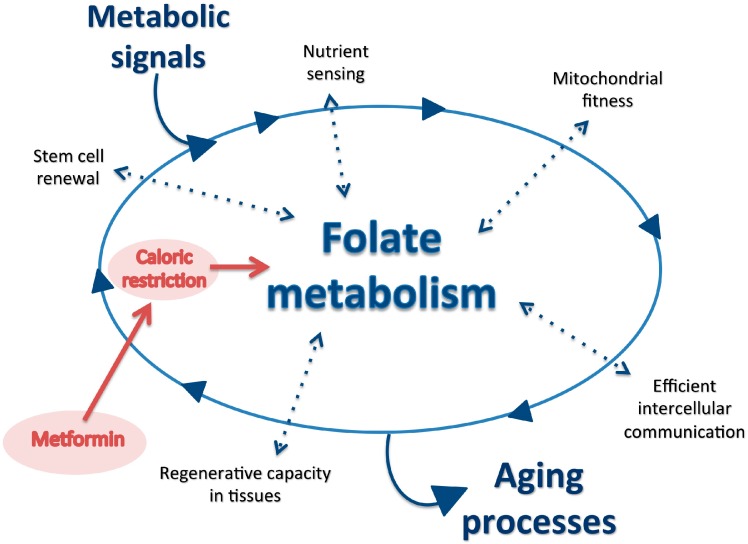
The effect of metformin on signaling pathways associated with nutrient excess. These effects are likely regulated by folate metabolism and represent direct effects on some of the hallmarks of tissue aging. It remains to be determined whether metformin can safely slow the development of age-related comorbidities in metabolic diseases.

Food restriction extends health and lifespan in some models [1], but what nutrients should be restricted and to what extent? Qualitative changes in the provision of dietary macronutrients induce metabolic and endocrine adaptations that are clinically relevant to the nutritional status of both, patients with low food availability and those with a persistent intake of excessive amounts of food [2]. In particular, the relationship between energy and one-carbon (1C) metabolism is extremely sensitive to food intake [3,4,5,6]. We envision that the inclusion of metformin in current strategies promoting metabolic fitness [7] is in accordance with the clinical response to dietary environment in disease states and the beneficial effects in metabolically vulnerable cells [8,9,10,11]. That is, it is important to revise what we can learn from metformin users and which are the potential implications. Metformin, as a calorie-restriction mimetic drug affecting mitochondrial function, integrates metabolic signals and the direct effect on folate metabolism may provide therapeutic clues [12,13,14,15]. We highlight the effects of metformin on signaling pathways associated with some of the hallmarks of aging and the likely beneficial effects in the pathogenesis of comorbidities associated with metabolic diseases (Figure 1). Whether metformin can be safely used with these new indications remains to be established.

Metformin is currently used exclusively in the treatment of type 2 diabetes mellitus (T2DM), but there is potential for additional indications in obesity, inflammatory disorders, cardiovascular diseases and cancer. For instance, in obese patients without diabetes, the weight loss effects of metformin are not inferior to those of a recently approved drug for obesity [16] and the antenatal administration of metformin during pregnancy reduces maternal weight gain without effects on neonatal outcomes [17,18,19]. Moreover, the Diabetes Prevention Program has found beneficial effects in diabetes prevention and a durable weight loss attributable to metformin [20,21,22]. Cardiovascular benefits are also likely, and, when compared to sulfonylurea or insulin therapy, metformin monotherapy is associated with a higher reduction in cardiovascular events [23,24]. In addition, the anti-inflammatory actions of metformin can be separated from its metabolic effects, and there is ongoing research assessing the role of metformin in the prevention and recurrence of breast cancer [25,26]. 

All these effects are crucial in preventing disease and promoting health. We discuss here the metabolic alterations connecting the metformin response and the function of essential nutrients, which emphasize that careful attention to diet might shape clinical strategies [27]. Our arguments are framed in the relationship between energy and one-carbon metabolism, reinforced by the mechanisms of action proposed for metformin.

## 2. One-Carbon Metabolism: Inputs and Outputs 

Folate coenzymes play a crucial role in health and disease and are present in virtually all organisms and cell types. Moreover, although controversial, some dietary arguments support the addition of folic acid or related compounds to common foods [28,29,30]. In humans, dietary folic acid is reduced to 7,8-dihydrofolate (DHF) and then to 5,6,7,8-tetrahydrofolate (THF) by dihydrofolate reductase (DHFR), initiating the folate cycle. Biochemical reactions converge here, using the ability of THF to carry 1C units in different oxidation states. Folate coenzymes contain poly-γ-glutamate tails attached to a p-aminobenzoic acid moiety by the activity of folylpolyglutamate synthetase (FPGS), an essential enzyme that regulates the distribution of different folate forms in cellular compartments and specific actions in cell proliferation pathways [31,32]. Serine, glycine and methionine are readily provided in the diet. Serine is oxidized in the mitochondria and transferred to THF by serine hydroxyl-methyl-transferase (SHMT), resulting in glycine and 5,10-methylene-THF (CH2-THF). Glycine may be incorporated directly into purine nucleotide bases or glutathione (GSH) and CH2-THF can be converted to 5-methyl-THF in a reaction catalyzed by methylenetetrahydrofolate reductase (MTHFR) or 10-formyl-THF according to cellular needs. Of note, serine and glycine may be synthesized de novo through glycolysis, aldol cleavage (from threonine, in some cells) or reactions involving demethylation (from choline, betaine, dimethylglycine and sarcosine) [31,32].

**Figure 2:**
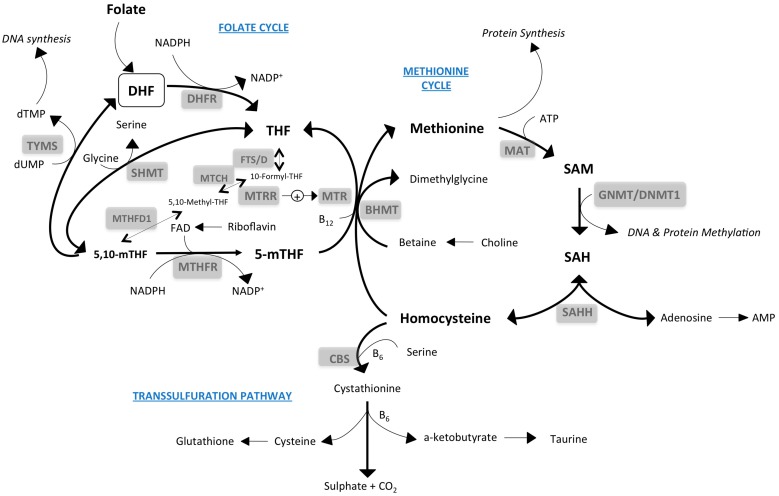
Metabolic pathways indicating the close dietary dependence in the folate cycle, methionine cycle and transsulfuration pathways. The role of B vitamins is paramount in regulating the expected complexities in relevant enzymes and circulating levels of metabolites. BHMT, betaine-homocysteine *S*-methyltransferase; CBS, cystathionine beta-synthase; DHFR, dihydrofolate reductase; DNMT, DNA methyltransferase; FAD, flavin adenine dinucleotide; GNMT, glycine *N*-methyltransferase; MAT, aminomethyltransferase; MTHFR, methylenetetrahydrofolate reductase; MTR, methyltransferase; SAH, S-adenosylhomocysteine; SAHH, S-adenosylhomocysteine hydrolase; SAM, S-adenosylmethionine; SHMT, serine hydroxymethyltransferase; THF, tetrahydrofolate; TYMS, thymidylate synthase; UDP, uridine diphosphate.

The folate cycle, closely linked to the methionine cycle, regulates the availability of methyl groups (CH3) through the sequential conversion of methionine to S-adenosylmethionine (SAM), S-adenosylhomocysteine (SAH) and homocysteine. Conversely, in the presence of 5-methyl-THF (5-mTHF) and methionine synthase, which requires vitamin B12 (cobalamin), methionine can be regenerated. Transmethylation reactions and intermediate metabolites are crucial to the synthesis of high-energy molecules, structural macromolecules, thymidine, and purines and to the maintenance of the cellular redox state and the transsulfuration pathway [33,34]. Dietary vitamins also communicate bioenergetics and 1C metabolism. For instance, the conversion of homocysteine to cysteine (transsulfuration pathway) and the synthesis of glutathione require vitamin B6 (pyridoxine) and vitamin B12 is essential (via the mitochondrial enzyme methylmalonyl-CoA mutase) to form succinyl-CoA, a key substrate of the citric acid cycle (Figure 2).

**Figure 3:**
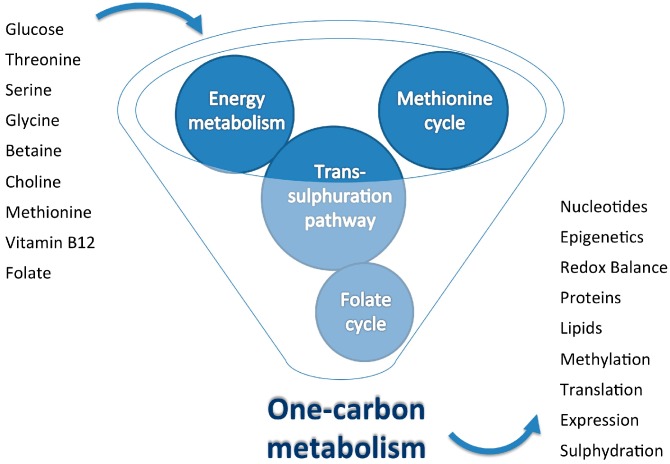
Schematic representation summarizing the importance of appropriate equilibrium in inputs and outputs of one-carbon metabolism. Excessive intake of a given nutrient influences the availability of other nutrients and can perturb metabolism with deleterious consequences. Understanding the events linking metabolism and diet-dependent pathways may provide crucial insight into their role in health and disease.

The cellular functions that depend on these micronutrients illustrate how important careful dietary intake is to avoid deficiencies and how difficult it is to establish clinically based data to be used in both the management of disease states and drug treatments. It may appear paradoxical, but diseases associated with excessive food intake may present deleterious effects that could result from excess in micronutrients [30]. The challenge is to achieve an adequate nutrient balance in the different groups within the general population. In this context, several complexities at the organismal level may be illustrative. For instance, the hepatic effects of diets deficient in choline and methionine are practically indistinguishable from those observed with methionine rich, high-fat, high-energy diets. In contrast, the balanced provision of methionine increases healthy lifespan in experimental models [35,36,37]. These nutritional observations exemplify the close dependence among regulatory pathways in energy and one-carbon metabolism and the critical importance of the equilibrium between inputs and outputs (Figure 2 and Figure 3).

## 3. The Lack of Redundancy between Cytoplasmatic and Mitochondrial One-Carbon Metabolism Affects Tissue-Specific Responses to Nutrients

Deficient or excessive intake of nutrients causes changes in the mitochondrial electron transport chain that model the organismal response to the environment. In human cells, the specific forms of folate cofactors and the extent of polyglutamylation differ in cytosolic and mitochondrial pools. Curiously, 1C metabolism is compartmentalized, and mechanisms controlling the flux of components through these compartments are strictly regulated ([38] and references therein). Whether mitochondrial dysfunction is a cause or a consequence of disease remains debatable, but it appears that mitochondria receive monoglutamate THF, which is polyglutamylated, charged with 1C units, and later released to the cytosol [39,40], ensuring the correct function of cellular 1C metabolism. 

Energy and one-carbon metabolism jointly integrate the cellular nutrient status. Reactions of glycine and serine are particularly important connecting nutrition, 1C metabolism and the effects of biguanides, as demonstrated in experimental models of glycine auxotrophy. Data in these models indicate that the cytoplasmic serine hydroxymethyltransferase (SHMT) isozyme is not essential, but glycine biosynthesis requires a different mitochondrial isozyme, which is not reversed by external addition of nutrients [41]. Moreover, in the absence of cytoplasmic SHMT, serine donates 1C units and formate flows normally to the cytoplasmic THF pool through the activity of mitochondrial methylene tetrahydrofolate dehydrogenase (MTHFD), a trifunctional folate-dependent enzyme with activity as a CH2-THF dehydrogenase, CH+-THF cyclohydrolase and 10-formyl-THF synthetase. Mice without these mitochondrial isozymes (MTHFD2/MTHFD2L) are not viable [42,43,44]. In addition, glycine is broken down by the exclusively mitochondrial glycine cleavage system (GCS) and in this reaction direction, the electrons are delivered to complex I of the mitochondrial respiratory chain [45]. Metformin affects mitochondrial biology at this point, but information on compensatory pathways is limited [8,31].

Understanding the transport processes between compartments and how these differences in cytoplasm and mitochondria control metabolic processes might provide a rationale for treating metabolic abnormalities. The strictly mitochondrial GCS activities support a model of multiple carrier-facilitated diffusion of metabolites between cytosol and mitochondria [30]. Because there is no methionine adenosyltransferase (MAT) activity in mitochondria, putative carriers are also necessary for the constant transport of SAM from the cytosol to the mitochondria [46]. The relationship between compartments might also explain the metabolism of choline, which is obtained primarily as phosphatidylcholine in the diet. In the liver, choline is a major source of methyl groups through conversion to betaine (*N*,*N*,*N*-trimethylglycine). The cytosolic betaine hydroxymethyltransferase (BHMT) generates methionine and *N*,*N*-dimethylglycine (DMG), but DMG absolutely requires the activity of mitochondrial dehydrogenase (DMGDH) to act as a 1C donor in mitochondria. Similarly, sarcosine (*N*-methylglycine) can be a 1C donor in the cytoplasm, but in the mitochondria, it requires sarcosine dehydrogenase (SDH) [47]. 

**Figure 4:**
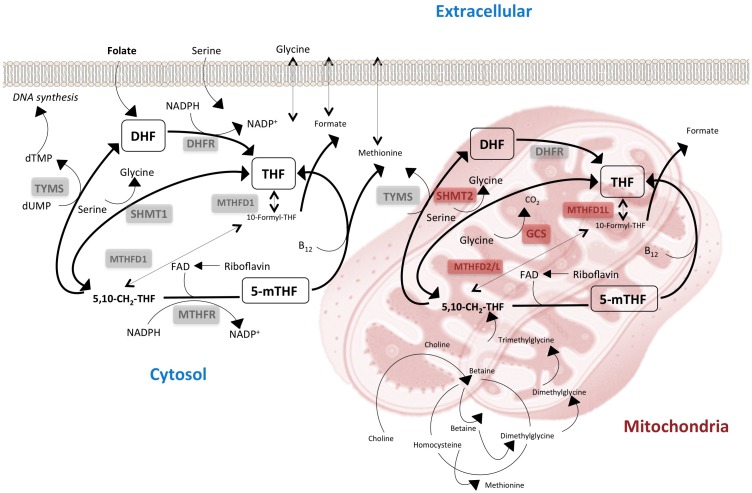
Mitochondrial and cytosolic pathways for generating and processing 1C units are separated but not redundant and depend on extracellular provision. Metabolically active cells may survive deficiencies in cytosolic isozymes but require the correct function of mitochondrial isozymes (marked in red). GCS, glycine cleavage system.

Collectively, these multiple regulatory steps indicate essential mechanisms for preventing disease. Experiments with genetically engineered mice, examining each step, are of limited value to understand human disease [48], but in humans there are associations between genetic variants and several diseases [49,50,51,52,53,54]. Similarly, available data suggest that mitochondrial 1C metabolism is critical for metabolic adaptations and cell survival in cancer and T cell-mediated (immune) pathologies [55,56,57]. T cell activation does not increase the expression of proteins related to glycolysis, pentose phosphate pathway or oxidative phosphorylation. In contrast, T cell activation produces new and specialized mitochondria characterized by the massive induction of enzymes involved in folate-mediated 1C metabolism [55], indicating that the mitochondrial and cytosolic pathways for generating and processing 1C units are not redundant. That is, cytosolic 1C metabolism is insufficient to support T cell proliferation, cancer cell immortality and excessive metabolic impact when mitochondrial 1C metabolism is impaired [31,58,59,60,61,62] (Figure 4). 

Mechanisms affecting energy and 1C metabolism are likely linked to cellular biosynthesis, redox maintenance and epigenetic status. Accurate information on the activities of the aforementioned enzymes in complex diseases might provide clues regarding how nutritional status or dietary and pharmacologic manipulations affect physiological regulation with evident therapeutic opportunities. 

## 4. The Importance of Drugs and Diets That Modulate Mitochondrial Activity 

Mitochondrial function, epigenetic signals and nutrient-sensing pathways are likely combined to promote health at both the cellular and the organismal levels [63,64]. Drugs and dietary regimens that directly modulate mitochondrial activity are promising [65]. 

Mitochondria are not only providers of energy but also of signaling units, and the existence of mitochondrial-derived peptides (MDPs) suggest the new concept of the existence of mitochondrial hormones and metabolic regulators [66,67,68]. Similar to metformin [12,69], targets of these MDPs include one-carbon metabolism, thiosulfate sulfurtransferase and AMP-activated protein kinase (AMPK), critical links between nutrients and health. Nutrient sensing is important for the distribution of energy, and changes in food intake alter metabolic strategies [70,71]. Notably, metformin activates AMPK, inducing changes in bioactive metabolites connected to transcriptional regulators through as yet undefined mechanisms [72,73] but probably including an interplay among food intake, metabolism and mTOR signaling in clinically relevant settings [74,75].

In particular, metabolic aspects in cancer are now a renewed source of potential therapeutic targets. Cells depleted of mitochondrial DNA identify metabolic vulnerabilities and illustrate that 1C metabolism, serine biosynthesis and transsulfuration are sensitive to mitochondrial dysfunction and stress [76]. Similarly, cancer cells without the ability to catalyze the conversion of isocitrate to α-ketoglutarate reveal that perturbing mitochondrial metabolism with metformin may help to kill cancer cells [77]. The challenge is to learn how to use cell- and tissue-specific variations in the mitochondrial management of 1C donors and how to interpret the clinical response to manipulations in nutrient availability. Several lines of evidence indicate that crosstalk between epigenetic signals and cellular metabolism may be a clinically useful field of research because it represents a mechanism to convert dietary-induced metabolic changes into stable patterns of altered gene expression [78,79,80,81].

## 5. Is Gene Expression Reprogrammed in Response to Metabolic and Dietary Stimuli Affecting One-Carbon Metabolism?

Whether epigenetic mechanisms are causally coupled to changes in metabolic phenotypes is a question posed by epidemiological studies examining extreme nutritional changes during fetal development [82,83,84,85]. It is not surprising, however, to find contradictory data because exposure windows and conditions are not controlled in documented “experiments” on long-term famine [82,83,84]. These studies do not reveal the contribution of specific nutrients, but studies in other cohorts associate defects in energy and one-carbon metabolism in pregnant women with future risk in offspring ([85] and references therein). Taken together, these data indicate that epigenetic modulation of metabolic pathways may promote adverse phenotypes in later life [86,87]. 

In the current context of excessive food intake, it is urgent to understand the potential implications of modifying epigenetic marks by nutrition and whether diet-induced changes in metabolic or phenotypic traits may affect future generations. Nutritional, pharmacological and metabolic signals induce epigenetic drift (i.e., metabolic states correlated with chromatin states), and several epigenetic marks influence the expression of neighboring genes through generations. It is apparently the feedback or combination among different epigenetic mechanisms that dynamically configures the chromatin landscape throughout life [88,89] but DNA methylation alone has strong mechanistic support to explain modifications by dietary changes. DNA methylation and certain metabolites generated by mitochondrial respiration interact with transcription factors, suggesting metabolo-epigenetic links between energy and one-carbon metabolism [90,91,92]. It is plausible that cellular transcriptional machinery and chromatin-associated proteins integrate inputs derived from food as part of the response of living organisms to continuous fluctuations in the availability of energy substrates.

Alterations in genes that encode enzymes affecting chromatin regulation, such as kinases, acetyltransferases, demethylases and methyltransferases, are common in dietary-related diseases [93]. These enzymes use cellular metabolites as sources of phosphate, acetyl or methyl groups, and interpret the metabolic state of a specific cell. For instance, the levels of SAM, a methyl donor, SAH and threonine alter methylation status through pathways involving acetyl-coA metabolism, and specific dietary restrictions cause transcriptional and metabolic responses [94,95]. We recently provided an example of how metformin-driven reduction of acetyl-CoA is sufficient to correct histone H3 acetylation, indicating that metformin regulates mitonuclear communication and the cellular epigenetic landscape. These data may result in knowledge that can be applied to metabolo-epigenetic strategies for prevention or therapy [96]. Future research will ascertain how dietary manipulations and synthetic epigenetic modifiers contribute to DNA methylation and how plastic the genome is to dietary changes. It is also important to establish what magnitude of metabolic stimuli is required to produce appreciable changes and whether epigenetic changes can be reversed. 

## 6. The Ability of Metformin to Target One-Carbon Metabolism: Perspectives in Clinical Practice Outcomes

The dietary inputs associated with the folate and methionine cycles support cellular integrity and health, but there is some risk in manipulating bioactive elements in the absence of well-proven associations between consumption and disease. This probably explains why nutritional manipulation is not incorporated into clinical practice against cancer, but we should not forget that the action of B vitamins led to the discovery of folate antagonists as a major class of cancer chemotherapy agents [97] and that restriction in some nutrients negatively affect tumor formation in mice [98]. 

The multi-faceted activity of metformin and its likely interconnected mechanisms of action are relevant to potential applications in diseases characterized by mitochondrial dysfunction, gene deregulation and failure in metabolic homeostasis [99,100,101,102,103]. The global metabolic impact of metformin and the generated energetic stress suggest a strong association with the overall management of food intake. Metformin stimulates glucose uptake by muscle, inhibits hepatic gluconeogenesis and stimulates AMPK through effects in NADH ubiquinone oxidoreductase, the first component of the electron transport chain. Other putative actions with dietary associations include AMPK-independent signaling through glucagon-dependent cyclic AMP and the inhibition of mitochondrial glycerophosphate dehydrogenase [103,104,105]. These findings on metformin action suggest an approach for treating metabolic diseases not restricted to the field of diabetes.

It could be argued that drugs reducing insulin levels may reduce cancer risk, and drugs that increase circulating insulin may increase cancer risk, but the effects of metformin in cancer cells seem specific, preventing the boost in glycolytic intermediates, decreasing citric acid cycle intermediates, and depleting the cellular glutathione content [106]. Moreover, metformin impairs one-carbon metabolism in a manner similar to drugs that target folate-related enzymes and have long been used to treat inflammatory diseases and cancer [8,12,106,107,108,109]. Efforts to clinically address this issue remain incomplete and difficult to interpret because heterogeneity in metformin response is considerable and only partially explained by genetic and nutritional factors. Metformin pharmacodynamics and pharmacokinetics have been reviewed recently ([110] and references therein). Current formulations have a bioavailability of ~50% (approximately 40% is absorbed in the duodenum and proximal jejunum, and ~10% in the ileum and colon). Unabsorbed drug is eliminated in the feces, and metformin circulates in the plasma unbound and without transformation until cleared by the kidneys. Metformin is a hydrophilic molecule requiring specific mechanisms of transport, primarily organic cation transporters (OCT). Briefly, plasma membrane monoamine transporter (PMAT) and OCT3 contribute to gastrointestinal uptake, and OCT1 may be responsible for transport into the interstitial fluid of the intestine. OCT1, OCT3 and multidrug and toxin extrusion proteins (MATE) are expressed on the basolateral membrane of hepatocytes. In the kidney, metformin is taken up into renal epithelial cells by OCT2 and excreted into the urine via MATE1 and MATE2. OCT1 and PMAT may contribute to metformin reabsorption. Genetic variants of these and other transporters may be important to explain the therapeutic action of metformin, the high inter-individual variability in gastrointestinal tolerance and drug–drug interactions [111,112,113,114]. It would be useful to ascertain whether these genetic variants could have predictive value in patients before they take the drug [115], but genetics alone frequently fail to explain phenotypes. In this context, we foresee that considering metformin for non-diabetic indications is an example of the value of applying metabolomics to useful clinical research. The next task is connecting energy and one-carbon metabolism to physiology, describing heterogeneous phenotypes and defining the role of nutrition in the response to drug treatment. 

## 7. The One-Carbon Cycle in Metformin Users and Potential Adverse Effects

We believe that metformin is best described as an “antimetabolite” drug, and, as such, several deleterious effects might be expected among long-term metformin users. Trials to ascertain these effects are scarce, and data are frequently contradictory, but people at risk of deficiency in one-carbon-related metabolites include vegans, vegetarians, pregnant women, breastfeeding women, the elderly and patients with anemia or poor renal function [116].

There is no evidence of risk in humans from taking metformin during pregnancy, but this issue requires attention [17]. Folate deficiency in metformin users is rare, but a decrease in serum and red blood cell (RBC) folate concentration is common [117]. It is difficult to ascertain the effect of folate fortification in some foods due to discrepancies in results [118] and the lack of data regarding folate bioavailability in metformin users [34,119]. Favoring the consumption of foods that are endogenously high in folates is probably reminiscent of decades using folates with cytotoxic agents, but this empirical practice requires caution. For instance, lessons from a prospective study in rural India indicate that normal/high folate concentrations associated with deficiencies in vitamin B12, attributable to a lacto-vegetarian diet combined with folic acid supplementation, may be associated with a higher risk of insulin resistance in the offspring [120]. This is relevant because metformin might facilitate the mechanism known as “methyl folate trap”, which acts on the regulation of the methionine/folate cycle and cysteine oxidation [121]. According to this concept, although only demonstrated in pernicious anemia, the cell would mistakenly interpret vitamin B12 deficiency as a lack of methionine, and will divert the remaining folate away from DNA biosynthesis towards the methylation of homocysteine to methionine, building up 5 methyl THF that the cell will not be able to use [121].

The relationship between vitamin B12 deficiency and metformin treatment has been studied since the beginning of the 1970s with some clinical confusion likely related to the extreme heterogeneity among studies [122,123,124]. Among metformin users, a slight reduction in serum vitamin B12 concentrations is common, but some studies have reported contradictory results indicating that metformin has no effect or even might improve vitamin B12 metabolism [125,126,127]. No mechanism has been proposed and the issue requires further research but there is probably no clinical relevance in these observations as discussed below. Moreover, the possible benefits of vitamin B12 supplements in metformin users have not been assayed and the notion of causality is complicated because diabetes and obesity are also associated with vitamin B12 reductions [128]. 

Metformin does not significantly increase blood lactate levels, but we are probably depriving some patients from potential benefits based on the putative risk of lactic acidosis [129]. After 70 years of real-world clinical experience, the incidence of lactic acidosis is mostly based in anecdotal reports. However, caution is important in individuals with reduced metformin clearance (i.e., poor renal function), reduced lactate clearance (i.e., impaired hepatic metabolism), and/or increased lactate production (i.e., sepsis or reduced tissue perfusion). Advanced age may also increase risk because of age-related decline in renal function and increased risk for acute renal failure (i.e., dehydration), drug–drug interactions and other medical conditions. Moreover, elderly patients with type 2 diabetes are independently at greater risk for hyperlactatemia and have a reduced threshold for the development of lactic acidosis in response to a secondary event [130]. 

The most frequent adverse effects are gastrointestinal in nature: diarrhea, nausea, and to a lesser extent, vomiting, flatulence or heartburn. Of note, in randomized controlled trials, similar effects are found in the placebo group, indicating potential bias [131]. Those patients without preexisting gastrointestinal conditions are apparently free of these effects, but it is not uncommon that some patients decline using metformin [112,113]. Metformin response and tolerance are intrinsically associated with the gut and the intake of nutrients [132]. Metformin could increase serum glucagon-like peptide 1 concentration by increasing its secretion from L cells, distributed throughout the intestine, and/or by reducing its breakdown by dipeptidyl peptidase-4 in the intestinal mucosa and portal system [133]. Curiously, serotonin and histamine release from the intestine are associated with similar effects (nausea, vomiting, increased gut motility and diarrhea). It is possible that metformin inhibits diamine oxidase, which is highly expressed in enterocytes and responsible for the metabolism of these gut peptides [134]. Metformin might also disrupt the enterohepatic circulation of bile salts, predominantly through reduced ileal absorption and osmotic effects facilitating diarrhea [135]. Finally, the gut microbiome is considered an environmental factor contributing to the development of metabolic diseases and a possible target of metformin. A reduction in butyrate-producing bacteria and an increase in opportunistic pathogens are common in type 2 diabetes and could potentially influence gastrointestinal tolerance [136,137,138]. 

## 8. Measuring the Impact of Folate-Related Deficiencies in the Clinical Setting: Potential for Targeted Metabolomics

Results from immunoassays in clinical laboratories are difficult to interpret and limited by the availability of reagents and automated biochemistry platforms. Methodological constraints, confounding factors, and poor inter-laboratory reproducibility are common pitfalls [139,140,141]. Consequently, the picture obtained when exploring folate metabolism is partial at best. For instance, total circulating B12 in serum is unreliable in some clinical conditions and practically useless as a method to detect true, functional B12 deficiency [142]. Most (80%) of B12 is bound in serum to haptocorrin, and a variable proportion (5%–20%) is bound to transcobalamin II and ready for tissue uptake (holotranscobalamin). Measuring in the same batch serum holotranscobalamin, homocysteine and/or methylmalonic acid can mitigate limitations [125,143]. Some pitfalls were made evident in a large cohort of participants enrolled in the REasons for Geographic and Racial Differences in Stroke (REGARDS) study [144]. The proportion of participants with low serum B12 concentration was exactly the same (2%; not clinically evident) in participants without diabetes and in long-term metformin users with diabetes. However, serum B12 concentrations were lower in metformin users than in those who did not use metformin. Curiously, metformin users were less likely to have taken multivitamins (6–25 μg of vitamin B12 per dose), and multivitamin users had a significantly higher serum B12 concentration compared to those who did not take multivitamins [144]. A longitudinal study to assess the impact of metformin is warranted, but it appears that laboratory biomarkers do not add significantly to clinical decisions and that dietary advice might contribute to better management of metformin users [145]. 

Similarly, measurement of serum folate with standard immunoassays is accompanied by possible errors in interpretation. For instance, obesity appears to be associated with decreased serum (measuring recent folate intake) but also with increased RBC folate (measuring a long term intake) concentrations compared with lean subjects. The association is plausible but the presence of obesity-associated metabolic disturbances hampers further interpretations [146]. It was recently clarified that folate measurements in serum and in RBC display similar performance in assessing folate status [147]. The use of both measures generates higher and unnecessary costs, but the RBC folate assay is less likely to provide falsely normal levels attributable to dietary behavior or recent supplements [147]. The observation that metformin is associated with a slight, but sometimes significant, raise in homocysteine and/or decrease in folate needs careful consideration, but there is no sufficient evidence to recommend folic acid supplements to metformin users [148,149]. 

It remains unknown how nutritional manipulations may affect the complex relationships among metabolites involved in the 1C cycle, but studies in women with seasonal variations in nutrient intake highlight the need to measure all metabolites and cofactors [150,151]. Analytical platforms should provide measurements of different folate species, especially 5-methyltetrahydrofolate as the active 1C donor [150,151]. Specifically, it is important to confirm data suggesting that levels of maternal one-carbon metabolites at conception influence DNA methylation in the early embryo and that offspring methylation correlates with the paternal somatic methylation pattern [152]. This effort implies better tools for measuring intermediate metabolites either side of the involved reactions [153,154]. Targeted metabolomics may help in pursuing a better interpretation. In this context, ultra-high pressure liquid chromatography coupled to an electrospray ionization source and a triple-quadrupole mass spectrometer is an affordable choice to quantitatively examine the methionine/folate bi-cyclic 1C metabolome [155]. This method has been used to explore the activation of methylogenesis in some models. This essential function of 1C metabolism provides a labile pool of methyl groups required for successfully establishing and maintaining the DNA methylation imprint [155]. A similar approach has been used to explore energy metabolism with a simple and rapid method based on gas chromatography coupled to quadrupole time-of-flight mass spectrometry and an electron impact source [156]. The accurate and simultaneous measurement of selected metabolites facilitates the understanding of metabolic responses to changing environmental factors and has the potential for searching quantitative biomarkers of disease and signals indicating the ability of drugs to restore cellular homeostasis [157]. In addition, this analytical approach may serve to assess the expected toxicity in potential applications for metformin employed in oncology at doses notably higher than those used chronically in the management of diabetes [8].

## 9. Conclusions

Metformin users may provide data on the effect of nutrients in health and disease. There is growing evidence demonstrating the multiple protective effects of metformin against obesity-associated diseases, a major challenge to global public health. Some findings support the idea that metformin mediates the mitochondrial response to excessive food intake and the effect of different micronutrients. In particular, the mechanism of action of metformin involves effects on both energy and one-carbon metabolism and suggests novel strategies that involve the combination of lifestyle modification with pharmacotherapy. The concept is more important in individuals whose risk factors are not reduced by dietary interventions and dietary regimens in metformin users may provide valuable information. We envision that several analytical approaches in the field of metabolomics can provide diagnostic indicators on multiple metabolic aspects and may ascertain the effects of nutrient intake. Accordingly, it is especially relevant to assess the role of significant nutrients, such as serine, glycine, methionine, folic acid or other B vitamins, affecting one-carbon metabolism. Efforts to repurpose metformin, the first choice as an oral treatment of type 2 diabetes, as an antimetabolite drug, reinforce the interest in understanding food and drug interactions and the expected toxic effects caused by a change in dose range. 

## References

[1] Mirzaei H., Di Biase S., Longo V.D. (2016). Dietary Interventions, Cardiovascular Aging, and Disease: Animal Models and Human Studies. Circ. Res..

[2] Hall K.D., Bemis T., Brychta R., Chen K.Y., Courville A., Crayner E.J., Goodwin S., Guo J., Howard L., Knuth N.D. (2015). Calorie for Calorie, Dietary Fat Restriction Results in More Body Fat Loss than Carbohydrate Restriction in People with Obesity. Cell Metab..

[3] Kim W., Woo H.D., Lee J., Choi I.J., Kim Y.W., Sung J., Kim J. (2016). Dietary folate, one-carbon metabolism-related genes, and gastric cancer risk in Korea. Mol. Nutr. Food Res..

[4] Martínez-Reyes I., Chandel N.S. (2014). Mitochondrial one-carbon metabolism maintains redox balance during hypoxia. Cancer Discov..

[5] Duncan T.M., Reed M.C., Nijhout H.F. (2013). A population model of folate-mediated one-carbon metabolism. Nutrients.

[6] Bailey L.B., Stover P.J., McNulty H., Fenech M.F., Gregory J.F., Mills J.L., Pfeiffer C.M., Fazili Z., Zhang M., Ueland P.M. (2015). Biomarkers of Nutrition for Development. J. Nutr..

[7] López-Otín C., Galluzzi L., Freije J.M., Madeo F., Kroemer G. (2016). Metabolic Control of Longevity. Cell.

[8] Menendez J.A., Quirantes-Piné R., Rodríguez-Gallego E., Cufí S., Corominas-Faja B., Cuyàs E., Bosch-Barrera J., Martin-Castillo B., Segura-Carretero A., Joven J. (2014). Oncobiguanides: Paracelsus’ law and nonconventional routes for administering diabetobiguanides for cancer treatment. Oncotarget.

[9] Pollak M. (2013). Potential applications for biguanides in oncology. J. Clin. Investig..

[10] Coperchini F., Leporati P., Rotondi M., Chiovato L. (2015). Expanding the therapeutic spectrum of metformin: From diabetes to cancer. J. Endocrinol. Investig..

[11] Pryor R., Cabreiro F. (2015). Repurposing metformin: An old drug with new tricks in its binding pockets. Biochem. J..

[12] Corominas-Faja B., Quirantes-Pine R., Oliveras-Ferraros C., Vazquez-Martin A., Cufi S., Martin-Castillo B., Micol V., Joven J., Segura-Carretero A., Menendez J.A. (2012). Metabolomic fingerprint reveals that metformin impairs one-carbon metabolism in a manner similar to the antifolate class of chemotherapy drugs. Aging.

[13] Novelle M.G., Ali A., Diéguez C., Bernier M., de Cabo R. (2016). Metformin: A Hopeful Promise in Aging Research. Cold Spring Harb. Perspect. Med..

[14] Carmona J.J., Michan S. (2016). Biology of Healthy Aging and Longevity. Rev. Investig. Clin..

[15] Cabreiro F., Au C., Leung K.Y., Vergara-Irigaray N., Cochemé H.M., Noori T., Weinkove D., Schuster E., Greene N.D., Gems D. (2013). Metformin retards aging in *C. elegans* by altering microbial folate and methionine metabolism. Cell.

[16] Domecq J.P., Prutsky G., Leppin A., Sonbol M.B., Altayar O., Undavalli C., Wang Z., Elraiyah T., Brito J.P., Mauck K.F. (2015). Clinical review: Drugs commonly associated with weight change: A systematic review and meta-analysis. J. Clin. Endocrinol. Metab..

[17] Syngelaki A., Nicolaides K.H., Balani J., Hyer S., Akolekar R., Kotecha R., Pastides A., Shehata H. (2016). Metformin versus Placebo in Obese Pregnant Women without Diabetes Mellitus. N. Engl. J. Med..

[18] Cassina M., Donà M., Di Gianantonio E., Litta P., Clementi M. (2014). First-trimester exposure to metformin and risk of birth defects: A systematic review and meta-analysis. Hum. Reprod. Update.

[19] Legro R.S., Arslanian S.A., Ehrmann D.A., Hoeger K.M., Murad M.H., Pasquali R., Welt C.K. (2013). Diagnosis and treatment of polycystic ovary syndrome: An Endocrine Society clinical practice guideline. J. Clin. Endocrinol. Metab..

[20] Knowler W.C., Fowler S.E., Hamman R.F., Christophi C.A., Hoffman H.J., Brenneman A.T., Brenneman A.T., Brown-Friday J.O., Goldberg R., Venditti E. (2009). 10-year follow-up of diabetes incidence and weight loss in the Diabetes Prevention Program Outcomes Study. Lancet.

[21] Hostalek U., Gwilt M., Hildemann S. (2015). Therapeutic Use of Metformin in Prediabetes and Diabetes Prevention. Drugs.

[22] Igel L.I., Sinha A., Saunders K.H., Apovian C.M., Vojta D., Aronne L.J. (2016). Metformin: An Old Therapy that Deserves a New Indication for the Treatment of Obesity. Curr. Atheroscler. Rep..

[23] Lamanna C., Monami M., Marchionni N., Mannucci E. (2011). Effect of metformin on cardiovascular events and mortality: A meta-analysis of randomized clinical trials. Diabetes Obes. Metab..

[24] DeFronzo R.A., Goodman A.M. (1995). Efficacy of metformin in patients with non-insulin-dependent diabetes mellitus. The Multicenter Metformin Study Group. N. Engl. J. Med..

[25] Cameron A.R., Morrison V.L., Levin D., Mohan M., Forteath C., Beall C., McNeilly A.D., Balfour D.J., Savinko T., Wong A.K. (2016). Anti-Inflammatory Effects of Metformin Irrespective of Diabetes Status. Circ. Res..

[26] Goodwin P.J. (2016). Obesity and Breast Cancer Outcomes: How Much Evidence Is Needed to Change Practice?. J. Clin. Oncol..

[27] Barzilai N., Crandall J.P., Kritchevsky S.B., Espeland M.A. (2016). Metformin as a Tool to Target Aging. Cell Metab..

[28] Allen L.H. (2016). Current Information Gaps in Micronutrient Research, Programs and Policy: How Can We Fill Them?. World Rev. Nutr. Diet.

[29] Bruins M.J., Kupka R., Zimmermann M.B., Lietz G., Engle-Stone R., Kraemer K. (2016). Maximizing the benefits and minimizing the risks of intervention programs to address micronutrient malnutrition: Symposium report. Matern. Child Nutr..

[30] Patel K.R., Sobczyńska-Malefora A. (2016). The adverse effects of an excessive folic acid intake. Eur. J. Clin. Nutr..

[31] Locasale J.W. (2013). Serine, glycine and one-carbon units: Cancer metabolism in full circle. Nat. Rev. Cancer.

[32] Mattaini K.R., Sullivan M.R., Vander Heiden M.G. (2016). The importance of serine metabolism in cancer. J. Cell Biol..

[33] Kopp M., Morisset R., Koehler P., Rychlik M. (2016). Stable Isotope Dilution Assays for Clinical Analyses of Folates and Other One-Carbon Metabolites: Application to Folate-Deficiency Studies. PLoS ONE.

[34] Mönch S., Netzel M., Netzel G., Ott U., Frank T., Rychlik M. (2015). Folate bioavailability from foods rich in folates assessed in a short term human study using stable isotope dilution assays. Food Funct..

[35] Mato J.M., Martinez-Chantar M.L., Lu S.C. (2008). Methionine metabolism and liver disease. Annu. Rev. Nutr..

[36] Pacana T., Cazanave S., Verdianelli A., Patel V., Min H.K., Mirshahi F., Quinlivan E., Sanyal A.J. (2015). Dysregulated Hepatic Methionine Metabolism Drives Homocysteine Elevation in Diet-Induced Nonalcoholic Fatty Liver Disease. PLoS ONE.

[37] Lee B.C., Kaya A., Gladyshev V.N. (2016). Methionine restriction and life-span control. Ann. N. Y. Acad. Sci..

[38] Tibbetts A.S., Appling D.R. (2010). Compartmentalization of Mammalian folate-mediated one-carbon metabolism. Annu. Rev. Nutr..

[39] Lin B.F., Huang R.F., Shane B. (1993). Regulation of folate and one-carbon metabolism in mammalian cells. III. Role of mitochondrial folylpoly-gamma-glutamate synthetase. J. Biol. Chem..

[40] Lawrence S.A., Titus S.A., Ferguson J., Heineman A.L., Taylor S.M., Moran R.G. (2014). Mammalian mitochondrial and cytosolic folylpolyglutamate synthetase maintain the subcellular compartmentalization of folates. J. Biol. Chem..

[41] McCarthy E.A., Titus S.A., Taylor S.M., Jackson-Cook C., Moran R.G. (2004). A mutation inactivating the mitochondrial inner membrane folate transporter creates a glycine requirement for survival of Chinese hamster cells. J. Biol. Chem..

[42] Christensen K.E., Patel H., Kuzmanov U., Mejia N.R., MacKenzie R.E. (2005). Disruption of the mthfd1 gene reveals a monofunctional 10-formyltetrahydrofolate synthetase in mammalian mitochondria. J. Biol. Chem..

[43] Field M.S., Kamynina E., Stover P.J. (2016). MTHFD1 regulates nuclear de novo thymidylate biosynthesis and genome stability. Biochimie.

[44] Giardina G., Brunotti P., Fiascarelli A., Cicalini A., Costa M.G., Buckle A.M., di Salvo M.L., Giorgi A., Marani M., Paone A. (2015). How pyridoxal 5′-phosphate differentially regulates human cytosolic and mitochondrial serine hydroxymethyltransferase oligomeric state. FEBS J..

[45] Hampson R.K., Barron L.L., Olson M.S. (1983). Regulation of the glycine cleavage system in isolated rat liver mitochondria. J. Biol. Chem..

[46] Horne D.W., Holloway R.S., Wagner C. (1997). Transport of S-adenosylmethionine in isolated rat liver mitochondria. Arch. Biochem. Biophys..

[47] O’Donoghue N., Sweeney T., Donagh R., Clarke K.J., Porter R.K. (2009). Control of choline oxidation in rat kidney mitochondria. Biochim. Biophys. Acta.

[48] Peng L., Dreumont N., Coelho D., Guéant J.L., Arnold C. (2016). Genetic animal models to decipher the pathogenic effects of vitamin B12 and folate deficiency. Biochimie.

[49] Cheng T.Y., Makar K.W., Neuhouser M.L., Miller J.W., Song X., Brown E.C., Beresford S.A., Zheng Y., Poole E.M., Galbraith R.L. (2015). Folate-mediated one-carbon metabolism genes and interactions with nutritional factors on colorectal cancer risk: Women’s Health Initiative Observational Study. Cancer.

[50] Pangilinan F., Molloy A.M., Mills J.L., Troendle J.F., Parle-McDermott A., Kay D.M., Browne M.L., McGrath E.C., Abaan H.O., Sutton M. (2014). Replication and exploratory analysis of 24 candidate risk polymorphisms for neural tube defects. BMC Med. Genet..

[51] Krishna S.M., Dear A., Craig J.M., Norman P.E., Golledge J. (2013). The potential role of homocysteine mediated DNA methylation and associated epigenetic changes in abdominal aortic aneurysm formation. Atherosclerosis.

[52] De Vilbiss E.A., Gardner R.M., Newschaffer C.J., Lee B.K. (2015). Maternal folate status as a risk factor for autism spectrum disorders: A review of existing evidence. Br. J. Nutr..

[53] Martorell L., Segués T., Folch G., Valero J., Joven J., Labad A., Vilella E. (2006). New variants in the mitochondrial genomes of schizophrenic patients. Eur. J. Hum. Genet..

[54] Vilella E., Virgos C., Murphy M., Martorell L., Valero J., Simó J.M., Joven J., Fernández-Ballart J., Labad A. (2005). Further evidence that hyperhomocysteinemia and methylenetetrahydrofolate reductase C677T and A1289C polymorphisms are not risk factors for schizophrenia. Prog. Neuropsychopharmacol. Biol. Psychiatry.

[55] Ron-Harel N., Santos D., Ghergurovich J.M., Sage P.T., Reddy A., Lovitch S.B., Dephoure N., Satterstrom F.K., Sheffer M., Spinelli J.B. (2016). Mitochondrial Biogenesis and Proteome Remodeling Promote One-Carbon Metabolism for T Cell Activation. Cell Metab..

[56] DeBerardinis R.J., Lum J.J., Hatzivassiliou G., Thompson C.B. (2008). The biology of cancer: Metabolic reprogramming fuels cell growth and proliferation. Cell Metab..

[57] Sullivan L.B., Gui D.Y., Hosios A.M., Bush L.N., Freinkman E., Vander Heiden M.G. (2015). Supporting aspartate biosynthesis is an essential function of respiration in proliferating cells. Cell.

[58] Fan J., Ye J., Kamphorst J.J., Shlomi T., Thompson C.B., Rabinowitz J.D. (2014). Quantitative flux analysis reveals folate-dependent NADPH production. Nature.

[59] Ye J., Fan J., Venneti S., Wan Y.W., Pawel B.R., Zhang J., Finley L.W., Lu C., Lindsten T., Cross J.R. (2014). Serine catabolism regulates mitochondrial redox control during hypoxia. Cancer Discov..

[60] Piskounova E., Agathocleous M., Murphy M.M., Hu Z., Huddlestun S.E., Zhao Z., Leitch A.M., Johnson T.M., DeBerardinis R.J., Morrison S.J. (2015). Oxidative stress inhibits distant metastasis by human melanoma cells. Nature.

[61] Jain M., Nilsson R., Sharma S., Madhusudhan N., Kitami T., Souza A.L., Kafri R., Kirschner M.W., Clish C.B., Mootha V.K. (2012). Metabolite profiling identifies a key role for glycine in rapid cancer cell proliferation. Science.

[62] Maddocks O.D., Berkers C.R., Mason S.M., Zheng L., Blyth K., Gottlieb E., Vousden K.H. (2013). Serine starvation induces stress and p53-dependent metabolic remodelling in cancer cells. Nature.

[63] Finkel T. (2015). The metabolic regulation of aging. Nat. Med..

[64] Sena L.A., Chandel N.S. (2012). Physiological roles of mitochondrial reactive oxygen species. Mol. Cell.

[65] Suliman H.B., Piantadosi C.A. (2016). Mitochondrial Quality Control as a Therapeutic Target. Pharmacol. Rev..

[66] Guo B., Zhai D., Cabezas E., Welsh K., Nouraini S., Satterthwait A.C., Reed J.C. (2003). Humanin peptide suppresses apoptosis by interfering with Bax activation. Nature.

[67] Lee C., Zeng J., Drew B.G., Sallam T., Martin-Montalvo A., Wan J., Kim S.J., Mehta H., Hevener A.L., de Cabo R. (2015). The mitochondrial-derived peptide MOTS-c promotes metabolic homeostasis and reduces obesity and insulin resistance. Cell Metab..

[68] Quirós P.M., Mottis A., Auwerx J. (2016). Mitonuclear communication in homeostasis and stress. Nat. Rev. Mol. Cell Biol..

[69] Morton N.M., Beltram J., Carter R.N., Michailidou Z., Gorjanc G., McFadden C., Barrios-Llerena M.E., Rodriguez-Cuenca S., Gibbins M.T., Aird R.E. (2016). Genetic identification of thiosulfate sulfurtransferase as an adipocyte-expressed antidiabetic target in mice selected for leanness. Nat. Med..

[70] Fitzgibbons T.P., Czech M.P. (2016). Emerging evidence for beneficial macrophage functions in atherosclerosis and obesity-induced insulin resistance. J. Mol. Med..

[71] Hardie D.G., Schaffer B.E., Brunet A. (2016). AMPK: An Energy-Sensing Pathway with Multiple Inputs and Outputs. Trends Cell Biol..

[72] Beltrán-Debón R., Rodríguez-Gallego E., Fernández-Arroyo S., Senan-Campos O., Massucci F.A., Hernández-Aguilera A., Sales-Pardo M., Guimerà R., Camps J., Menendez J.A. (2015). The acute impact of polyphenols from Hibiscus sabdariffa in metabolic homeostasis: An approach combining metabolomics and gene-expression analyses. Food Funct..

[73] Liu C., Wu J., Zhu J., Kuei C., Yu J., Shelton J., Sutton S.W., Li X., Yun S.J., Mirzadegan T. (2009). Lactate inhibits lipolysis in fat cells through activation of an orphan G-protein-coupled receptor, GPR81. J. Biol. Chem..

[74] Haas R., Cucchi D., Smith J., Pucino V., Macdougall C.E., Mauro C. (2016). Intermediates of Metabolism: From Bystanders to Signalling Molecules. Trends Biochem. Sci..

[75] Jha A.K., Huang S.C., Sergushichev A., Lampropoulou V., Ivanova Y., Loginicheva E., Chmielewski K., Stewart K.M., Ashall J., Everts B. (2015). Network integration of parallel metabolic and transcriptional data reveals metabolic modules that regulate macrophage polarization. Immunity.

[76] Bao X.R., Ong S.E., Goldberger O., Peng J., Sharma R., Thompson D.A., Vafai S.B., Cox A.G., Marutani E., Ichinose F. (2016). Mitochondrial dysfunction remodels one-carbon metabolism in human cells. eLife.

[77] Cuyàs E., Fernández-Arroyo S., Corominas-Faja B., Rodríguez-Gallego E., Bosch-Barrera J., Martin-Castillo B., de Llorens R., Joven J., Menendez J.A. (2015). Oncometabolic mutation IDH1 R132H confers a metformin-hypersensitive phenotype. Oncotarget.

[78] Katada S., Imhof A., Sassone-Corsi P. (2012). Connecting threads: Epigenetics and metabolism. Cell.

[79] Huypens P., Sass S., Wu M., Dyckhoff D., Tschöp M., Theis F., Marschall S., Hrabe de Angelis M., Beckers J. (2016). Epigenetic germline inheritance of diet-induced obesity and insulin resistance. Nat. Genet..

[80] Öst A., Lempradl A., Casas E., Weigert M., Tiko T., Deniz M., Pantano L., Boenisch U., Itskow P.M., Stoeckius M. (2014). Paternal diet defines offspring chromatin state and intergenerational obesity. Cell.

[81] Foster G.D., Wyatt H.R., Hill J.O., Makris A.P., Rosenbaum D.L., Brill C., Stein R.I., Mohammed B.S., Miller B., Rader D.J. (2010). Weight and metabolic outcomes after 2 years on a low-carbohydrate versus low-fat diet: A randomized trial. Ann. Intern. Med..

[82] Painter R.C., Roseboom T.J., Bleker O.P. (2005). Prenatal exposure to the Dutch famine and disease in later life: An overview. Reprod. Toxicol..

[83] Li Y., He Y., Qi L., Jaddoe V.W., Feskens E.J., Yang X., Ma G., Hu F.N. (2010). Exposure to the Chinese famine in early life and the risk of hyperglycemia and type 2 diabetes in adulthood. Diabetes.

[84] Stanner S.A., Bulmer K., Andrès C., Lantseva O.E., Borodina V., Poteen V.V., Yudkin J.S. (1997). Does malnutrition in utero determine diabetes and coronary heart disease in adulthood? Results from the Leningrad siege study, a cross sectional study. BMJ.

[85] Finer S., Saravanan P., Hitman G., Yajnik C. (2014). The role of the one-carbon cycle in the developmental origins of Type 2 diabetes and obesity. Diabet. Med..

[86] Tobi E.W., Goeman J.J., Monajemi R., Gu H., Putter H., Zhang Y., Slieker R.C., Stok A.P., Thijssen P.E., Müller F. (2014). DNA methylation signatures link prenatal famine exposure to growth and metabolism. Nat. Commun..

[87] Hernández-Aguilera A., Fernández-Arroyo S., Cuyàs E., Luciano-Mateo F., Cabre N., Camps J., Lopez-Miranda J., Menendez J.A., Joven J. (2016). Epigenetics and nutrition-related epidemics of metabolic diseases: Current perspectives and challenges. Food. Chem. Toxicol..

[88] Klosin A., Lehner B. (2016). Mechanisms, timescales and principles of trans-generational epigenetic inheritance in animals. Curr. Opin. Genet. Dev..

[89] Fontana L., Partridge L. (2015). Promoting health and longevity through diet: From model organisms to humans. Cell.

[90] Martinez-Outschoorn U.E., Peiris-Pagés M., Pestell R.G., Sotgia F., Lisanti M.P. (2016). Cancer metabolism: A therapeutic perspective. Nat. Rev. Clin. Oncol..

[91] Maddocks O.D., Labuschagne C.F., Adams P.D., Vousden K.H. (2016). Serine metabolism supports the methionine cycle and DNA/RNA methylation through de novo ATP Synthesis in Cancer Cells. Mol. Cell.

[92] Mentch S.J., Locasale J.W. (2016). One-carbon metabolism and epigenetics: Understanding the specificity. Ann. N. Y. Acad. Sci..

[93] Dawson M.A., Kouzarides T. (2012). Cancer epigenetics: From mechanism to therapy. Cell.

[94] Fang D., Gan H., Lee J.H., Han J., Wang Z., Riester S.M., Jin L., Chen J., Zhou H., Wang J. (2016). The histone H3.3K36M mutation reprograms the epigenome of chondroblastomas. Science.

[95] Orgeron M.L., Stone K.P., Wanders D., Cortez C.C., Van N.T., Gettys T.W. (2014). The impact of dietary methionine restriction on biomarkers of metabolic health. Prog. Mol. Biol. Transl. Sci..

[96] Cuyàs E., Fernández-Arroyo S., Joven J., Menendez J.A. (2016). Metformin targets histone acetylation in cancer-prone epithelial cells. Cell Cycle.

[97] Farber S., Diamond L.K. (1948). Temporary remissions in acute leukemia in children produced by folic acid antagonist, 4-aminopteroyl-glutamic acid. N. Engl. J. Med..

[98] Tannenbaum A. (1945). The dependence of tumor formation on the composition of the calorie- restricted diet as well as on the degree of restriction. Cancer Res..

[99] Heckman-Stoddard B.M., Gandini S., Puntoni M., Dunn B.K., De Censi A., Szabo E. (2016). Repurposing old drugs to chemoprevention: The case of metformin. Semin. Oncol..

[100] Marini C., Bianchi G., Buschiazzo A., Ravera S., Martella R., Bottoni G., Petretto A., Emionite L., Monteverde E., Capitanio S. (2016). Divergent targets of glycolysis and oxidative phosphorylation result in additive effects of metformin and starvation in colon and breast cancer. Sci. Rep..

[101] Menendez J.A., Oliveras-Ferraros C., Cufí S., Corominas-Faja B., Joven J., Martin-Castillo B. (2012). Vazquez-Martin, A. Metformin is synthetically lethal with glucose withdrawal in cancer cells. Cell Cycle.

[102] Van Wijk J.P., de Koning E.J., Cabezas M.C., op’t Roodt J., Joven J., Rabelink T.J., Hoepelman A.I. (2005). Comparison of rosiglitazone and metformin for treating HIV lipodystrophy: A randomized trial. Ann. Intern. Med..

[103] Hawley S.A., Ross F.A., Chevtzoff C., Green K.A., Evans A., Fogarty S., Towler M.C., Brown L.J., Ogunbayo O.A., Evans A.M. (2010). Use of cells expressing gamma subunit variants to identify diverse mechanisms of AMPK activation. Cell Metab..

[104] Zhou G., Myers R., Li Y., Chen Y., Shen X., Fenyk-Melody J., Wu M., Ventre J., Doebber T., Fujii N. (2001). Role of AMP-activated protein kinase in mechanism of metformin action. J. Clin. Investig..

[105] Madiraju A.K., Erion D.M., Rahimi Y., Zhang X.M., Braddock D.T., Albright R.A., Prigaro B.J., Wood J.L., Bhanot S., MacDonald M.J. (2014). Metformin suppresses gluconeogenesis by inhibiting mitochondrial glycerophosphate dehydrogenase. Nature.

[106] Menendez J.A., Cufí S., Oliveras-Ferraros C., Martin-Castillo B., Joven J., Vellon L., Vazquez-Martín A. (2011). Metformin and the ATM DNA damage response (DDR): Accelerating the onset of stress-induced senescence to boost protection against cancer. Aging.

[107] Menendez J.A., Joven J. (2012). One-carbon metabolism: An aging-cancer crossroad for the gerosuppressant metformin. Aging.

[108] Menendez J.A., Martin-Castillo B., Joven J. (2016). Metformin and cancer: Quo vadis et cui bono?. Oncotarget.

[109] Municio C., Soler-Palacios B., Estrada-Capetillo L., Benguria A., Dopazo A., García-Lorenzo E., Fernández-Arroyo S., Joven J., Miranda-Carús M.E., González-Álvaro I. (2016). Methotrexate selectively targets human proinflammatory macrophages through a thymidylate synthase/p53 axis. Ann. Rheum. Dis..

[110] Rena G., Pearson E.R., Sakamoto K. (2013). Molecular mechanism of action of metformin: Old or new insights?. Diabetologia.

[111] Shu Y., Sheardown S.A., Brown C., Owen R.P., Zhang S., Castro R.A., Ianculescu A.G., Yue L., Lo J.C., Burchard E.G. (2007). Effect of genetic variation in the organic cation transporter 1 (OCT1) on metformin action. J. Clin. Investig..

[112] Dujic T., Zhou K., Donnelly L.A., Tavendale R., Palmer C.N., Pearson E.R. (2015). Association of organic cation transporter 1 with intolerance to metformin in type 2 diabetes: A GoDARTS study. Diabetes.

[113] Dujic T., Causevic A., Bego T., Malenica M., Velija-Asimi Z., Pearson E.R., Semiz S. (2016). Organic cation transporter 1 variants and gastrointestinal side effects of metformin in patients with type 2 diabetes. Diabet. Med..

[114] Zhou K., Bellenguez C., Spencer C.C., Bennett A.J., Coleman R.L., Tavendale R., Hawley S.A., Donnelly L.A., Schofield C., Groves C.J. (2011). Common variants near ATM are associated with glycemic response to metformin in type 2 diabetes. Nat. Genet..

[115] Scheen A.J. (2014). Personalising metformin therapy: A clinician’s perspective. Lancet Diabetes Endocrinol..

[116] Ter Borg S., de Groot L.C., Mijnarends D.M., de Vries J.H., Verlaan S., Meijboom S., Luiking Y.C., Schols J.M. (2016). Differences in Nutrient Intake and Biochemical Nutrient Status Between Sarcopenic and Nonsarcopenic Older Adults-Results From the Maastricht Sarcopenia Study. J. Am. Med. Dir. Assoc..

[117] Malaguarnera G., Gagliano C., Salomone S., Giordano M., Bucolo C., Pappalardo A., Drago F., Caraci F., Avitabile T., Motta M. (2015). Folate status in type 2 diabetic patients with and without retinopathy. Clin. Ophthalmol..

[118] Mudryj A.N., de Groh M., Aukema H.M., Yu N. (2016). Folate intakes from diet and supplements may place certain Canadians at risk for folic acid toxicity. Br. J. Nutr..

[119] Danenberg P.V., Gustavsson B., Johnston P., Lindberg P., Moser R., Odin E., Peters G.J., Petrelli N. (2016). Folates as adjuvants to anticancer agents: Chemical rationale and mechanism of action. Crit. Rev. Oncol. Hematol..

[120] Yajnik C.S., Deshpande S.S., Jackson A.A., Refsum H., Rao S., Fisher D.J., Bhat D.S., Naik S.S., Coyaki K.J., Joglekar C.V. (2008). Vitamin B12 and folate concentrations during pregnancy and insulin resistance in the offspring: The Pune Maternal Nutrition Study. Diabetologia.

[121] Scott J.M., Weir D.G. (1981). The methyl folate trap. A physiological response in man to prevent methyl group deficiency in kwashiorkor (methionine deficiency) and an explanation for folic-acid induced exacerbation of subacute combined degeneration in pernicious anaemia. Lancet.

[122] Stowers J.M., Smith O.A. (1971). Vitamin B12 and metformin. BMJ.

[123] Ting R.Z., Szeto C.C., Chan M.H., Ma K.K., Chow K.M. (2006). Risk factors of vitamin B12 deficiency in patients receiving metformin. Arch. Intern. Med..

[124] Liu Q., Li S., Quan H., Li J. (2014). Vitamin B12 status in metformin treated patients: Systematic review. PLoS ONE.

[125] Obeid R., Jung J., Falk J., Herrmann W., Geisel J., Friesenhahn-Ochs B., Lammerts F., Fassbender K., Kostopoulos P. (2013). Serum vitamin B12 not reflecting vitamin B12 status in patients with type 2 diabetes. Biochimie.

[126] Greibe E., Trolle B., Bor M.V., Lauszus F.F., Nexo E. (2013). Metformin lowers serum cobalamin without changing other markers of cobalamin status: A study on women with polycystic ovary syndrome. Nutrients.

[127] Leung S., Mattman A., Snyder F., Kassam R., Meneilly G., Nexo E. (2010). Metformin induces reductions in plasma cobalamin and haptocorrin bound cobalamin levels in elderly diabetic patients. Clin. Biochem..

[128] Ahmed M.A. (2016). Metformin and Vitamin B12 Deficiency: Where Do We Stand?. J. Pharm. Pharm. Sci..

[129] DeFronzo R., Fleming G.A., Chen K., Bicsak T.A. (2016). Metformin-associated lactic acidosis: Current perspectives on causes and risk. Metabolism.

[130] Pernicova I., Korbonits M. (2014). Metformin—Mode of action and clinical implications for diabetes and cancer. Nat. Rev. Endocrinol..

[131] De Jager J., Kooy A., Lehert P., Wulffelé M.G., van der Kolk J., Bets D., Verburg J., Donker A.J., Stehouwer C.D. (2010). Long term treatment with metformin in patients with type 2 diabetes and risk of vitamin B-12 deficiency: Randomised placebo controlled trial. BMJ.

[132] McCreight L.J., Bailey C.J., Pearson E.R. (2016). Metformin and the gastrointestinal tract. Diabetologia.

[133] Wu T., Thazhath S.S., Bound M.J., Jones K.L., Horowitz M., Rayner C.K. (2014). Mechanism of increase in plasma intact GLP-1 by metformin in type 2 diabetes: Stimulation of GLP-1 secretion or reduction in plasma DPP-4 activity?. Diabetes Res. Clin. Pract..

[134] Yee S.W., Lin L., Merski M., Keiser M.J., Gupta A., Zhang Y., Chien H.C., Shoichet B.K., Giacomini K.M. (2015). Prediction and validation of enzyme and transporter off-targets for metformin. J. Pharmacokinet. Pharmacodyn..

[135] Scarpello J.H., Hodgson E., Howlett H.C. (1998). Effect of metformin on bile salt circulation and intestinal motility in type 2 diabetes mellitus. Diabet. Med..

[136] Forslund K., Hildebrand F., Nielsen T., Falony G., Le Chatelier E., Sunagawa S., Prifti E., Vieira-Silva S., Gudmundsdottir V., Krogh Pedersen H. (2015). Disentangling type 2 diabetes and metformin treatment signatures in the human gut microbiota. Nature.

[137] Burton J.H., Johnson M., Johnson J., Hsia D.S., Greenway F.L., Heiman M.L. (2015). Addition of a gastrointestinal microbiome modulator to metformin improves metformin tolerance and fasting glucose levels. J. Diabetes Sci. Technol..

[138] Mardinoglu A., Boren J., Smith U. (2016). Confounding Effects of Metformin on the Human Gut Microbiome in Type 2 Diabetes. Cell Metab..

[139] Bhalerao K.D., Lee S.C., Soboyejo W.O., Soboyejo A.B. (2007). A folic acid-based functionalized surface for biosensor systems. J. Mater. Sci. Mater. Med..

[140] Armbruster D.A., Alexander D.B. (2006). Sample to sample carryover: A source of analytical laboratory error and its relevance to integrated clinical chemistry/immunoassay systems. Clin. Chim. Acta.

[141] Bertran N., Camps J., Fernández-Ballart J., Murphy M.M., Arija V., Ferré N., Tous M., Joven J. (2005). Evaluation of a high-sensitivity turbidimetric immunoassay for serum C-reactive protein: Application to the study of longitudinal changes throughout normal pregnancy. Clin. Chem. Lab. Med..

[142] Wainwright P., Narayanan S., Cook P. (2015). False-normal vitamin B12 results in a patient with pernicious anaemia. Clin. Biochem..

[143] Harrington D.J. (2016). Holotranscobalamin: In the middle of difficultly lies opportunity. Clin. Chem. Lab. Med..

[144] Kancherla V., Garn J.V., Zakai N.A., Williamson R.S., Cashion W.T., Odewole O., Judd S.E., Oakley G.P. (2016). Multivitamin Use and Serum Vitamin B12 Concentrations in Older-Adult Metformin Users in REGARDS, 2003–2007. PLoS ONE.

[145] Russo G.T., Giandalia A., Romeo E.L., Scarcella C., Gambadoro N., Zingale R., Forte F., Perdichizzi G., Alibrandi A., Cucinotta D. (2016). Diabetic neuropathy is not associated with homocysteine, folate, vitamin B12 levels, and MTHFR C677T mutation in type 2 diabetic outpatients taking metformin. J. Endocrinol. Investig..

[146] Bird J.K., Ronnenberg A.G., Choi S.W., Du F., Mason J.B., Liu Z. (2015). Obesity is associated with increased red blood cell folate despite lower dietary intakes and serum concentrations. J. Nutr..

[147] Denimal D., Brindisi M.C., Lemaire S., Duvillard L. (2016). Assessment of Folate Status in Obese Patients: Should We Measure Folate in Serum or in Red Blood Cells?. Obes. Surg..

[148] Yetley E.A., Johnson C.L. (2011). Folate and vitamin B-12 biomarkers in NHANES: History of their measurement and use. Am. J. Clin. Nutr..

[149] Zhang Q., Li S., Li L., Li Q., Ren K., Sun X., Li J. (2016). Metformin treatment and homocysteine: A systematic review and meta-analysis of randomized controlled trials. Nutrients.

[150] Pfeiffer C.M., Sternberg M.R., Fazili Z., Lacher D.A., Zhang M., Johnson C.L., Hamner H.C., Bailey R.L., Rader J.I., Yamini S. (2015). Folate status and concentrations of serum folate forms in the US population: National Health and Nutrition Examination Survey 2011–2012. Br. J. Nutr..

[151] Dominguez-Salas P., Moore S.E., Cole D., da Costa K.A., Cox S.E., Dyer R.A., Fuldord A.J., Innis S.M., Waterland R.A., Zeisel, S.H. (2013). DNA methylation potential: Dietary intake and blood concentrations of one-carbon metabolites and cofactors in rural African women. Am. J. Clin. Nutr..

[152] Kühnen P., Handke D., Waterland R.A., Hennig B.J., Silver M., Fulford A.J., Dominguez-Salas P., Moore S.E., Prentice A.M., Spranger J. (2016). Interindividual Variation in DNA Methylation at a Putative POMC Metastable Epiallele Is Associated with Obesity. Cell Metab..

[153] Eicher-Miller H.A., Fulgoni V.L., Keast D.R. (2015). Processed Food Contributions to Energy and Nutrient Intake Differ among US Children by Race/Ethnicity. Nutrients.

[154] Colditz G.A. (2010). Overview of the epidemiology methods and applications: Strengths and limitations of observational study designs. Crit. Rev. Food. Sci. Nutr..

[155] Fernández-Arroyo S., Cuyàs E., Bosch-Barrera J., Alarcón T., Joven J., Menendez J.A. (2016). Activation of the methylation cycle in cells reprogrammed into a stem cell-like state. Oncoscience.

[156] Riera-Borrull M., Rodríguez-Gallego E., Hernández-Aguilera A., Luciano F., Ras R., Cuyàs E., Camps J., Segura-Carretero A., Menendez J.A., Joven J. (2016). Exploring the Process of Energy Generation in Pathophysiology by Targeted Metabolomics: Performance of a Simple and Quantitative Method. J. Am. Soc. Mass. Spectrom..

[157] Liu X., Romero I.L., Litchfield L.M., Lengyel E., Locasale J.W. (2016). Metformin Targets Central Carbon Metabolism and Reveals Mitochondrial Requirements in Human Cancers. Cell Metab..

